# Antibacterial activity of alkyl gallates is a combination of direct targeting of FtsZ and permeabilization of bacterial membranes

**DOI:** 10.3389/fmicb.2015.00390

**Published:** 2015-04-29

**Authors:** Ewa Król, Anabela de Sousa Borges, Isabel da Silva, Carlos R. Polaquini, Luis O. Regasini, Henrique Ferreira, Dirk-Jan Scheffers

**Affiliations:** ^1^Department of Molecular Microbiology, Groningen Biomolecular Sciences and Biotechnology Institute, University of GroningenGroningen, Netherlands; ^2^Departamento de Bioquímica e Microbiologia, Instituto de Biociências, Universidade Estadual PaulistaRio Claro, Brazil; ^3^Departamento de Química e Ciências Ambientais, Instituto de Biociências, Letras e Ciências Exatas, Universidade Estadual PaulistaSão José do Rio Preto, Brazil

**Keywords:** antibiotics, natural products, cell division, citrus canker, *Xanthomonas citri*, *Bacillus subtilis*

## Abstract

Alkyl gallates are compounds with reported antibacterial activity. One of the modes of action is binding of the alkyl gallates to the bacterial membrane and interference with membrane integrity. However, alkyl gallates also cause cell elongation and disruption of cell division in the important plant pathogen *Xanthomonas citri* subsp. *citri*, suggesting that cell division proteins may be targeted by alkyl gallates. Here, we use *Bacillus subtilis* and purified *B. subtilis* FtsZ to demonstrate that FtsZ is a direct target of alkyl gallates. Alkyl gallates disrupt the FtsZ-ring *in vivo*, and cause cell elongation. *In vitro*, alkyl gallates bind with high affinity to FtsZ, causing it to cluster and lose its capacity to polymerize. The activities of a homologous series of alkyl gallates with alkyl side chain lengths ranging from five to eight carbons (C_5_–C_8_) were compared and *heptyl* gallate was found to be the most potent FtsZ inhibitor. Next to the direct effect on FtsZ, alkyl gallates also target *B. subtilis* membrane integrity—however the observed anti-FtsZ activity is not a secondary effect of the disruption of membrane integrity. We propose that both modes of action, membrane disruption and anti-FtsZ activity, contribute to the antibacterial activity of the alkyl gallates. We propose that *heptyl* gallate is a promising hit for the further development of antibacterials that specifically target FtsZ.

## Introduction

The use of plants as sources of antimicrobial agents has a long history (Abreu et al., 2012). One group of plant compounds known for their antimicrobial activities are alkyl gallates, esters of gallic acid, a main product of tannin hydrolysis. Alkyl gallates have been shown to exhibit a broad spectrum of antibacterial activities against both Gram-positive and Gram-negative bacteria, including foodborne *Salmonella* (Kubo et al., 2002a), Methicillin Resistant *Staphylococcus aureus* (MRSA) (Kubo et al., 2002b; Shibata et al., 2005), *Bacillus subtilis* (Kubo et al., 2004), the plant pathogen *Xanthomonas citri* subsp. *citri* (Silva et al., 2013), and various others (Kubo et al., 2002a,b, 2003). Alkyl gallates with varying alkyl side chain lengths (C_1_–C_14_), have been studied as antibacterial agents alone or as modulators of the activities of β-lactams against MRSA (Kubo et al., 2002b, 2003, 2004; Shibata et al., 2005; Silva et al., 2013), a common cause of bloodstream infections in hospitals and healthcare facilities worldwide. The hydrolysis of alkyl gallates produces gallic acid and the corresponding alcohols (or alkanols), which both are common components in many plants.

Although the alkyl gallates have a head-and-tail structure similar to alkanols, suggesting that their antibacterial mode of action may be as surface-active agents affecting membrane integrity (Kubo et al., 2002b; Takai et al., 2011), Kubo et al. proposed that their antimicrobial activity is unlikely to be due to their surfactant property (Kubo et al., 2002a,b, 2003, 2004). Recently, we showed that alkyl gallates are active against *X. citri* subsp *citri* (Xac), an important plant pathogen that is the causative agent of citrus canker, one of the most damaging infections in citriculture. Pentyl, hexyl, heptyl, and octyl gallate treatment resulted in elongated Xac cells and disruption of the cell division machinery in this bacterium (Silva et al., 2013). Octyl gallate has been reported to exhibit bactericidal activity only against dividing and exponentially growing cells of *B. subtilis* but did not affect the viability of cells in the stationary phase (Kubo et al., 2004). Taken together, these results indicate that alkyl gallates may affect functions associated with cell division in Gram-positive and Gram-negative bacteria (Kubo et al., 2004; Silva et al., 2013).

Cell division is a relatively novel target for antibacterial drugs (Huang et al., 2007; Lock and Harry, 2008; Kapoor and Panda, 2009). Division is an essential process, which starts with the polymerization of the highly conserved cytoplasmic protein FtsZ in the middle of the cell leading to the formation of the so-called Z-ring (Adams and Errington, 2009; Erickson et al., 2010). After assembly of the Z-ring, several other proteins are recruited to mid-cell, resulting in a complex called the divisome, which carries out cell division at the correct time and place in the cell. Formation of the divisome depends on the assembly of FtsZ. FtsZ belongs to the tubulin family of cytoskeletal GTPases. The binding of GTP to FtsZ promotes the assembly of FtsZ monomers into long filaments *in vitro* (Kapoor and Panda, 2009). FtsZ is conserved among bacteria and is essential for cell viability, making it a potential target for new antibiotic discovery (Lock and Harry, 2008; Kapoor and Panda, 2009). Several natural, synthetic and semi-synthetic compounds were identified as inhibitors of FtsZ from Gram-positive and Gram-negative bacteria (Beuria et al., 2005; Lock and Harry, 2008; Rai et al., 2008; Andreu et al., 2010; Hemaiswarya et al., 2011; Anderson et al., 2012; Keffer et al., 2013).

To establish whether alkyl gallates indeed target bacterial cell division, we characterized the mode of action of alkyl gallates with a side chain length ranging from five to eight carbons (Table 1) in more detail, using *B. subtilis* as a model. We show that *B. subtilis* FtsZ is a target for these esters and that some of these compounds bind FtsZ with high affinity, resulting in protein cluster formation and disruption of FtsZ structures *in vitro* and *in vivo*. Additionally, the alkyl gallates interfere with the stability of the cell membrane. FtsZ binding and inhibition and membrane integrity are differently affected based on the alkyl chain length. Our results indicate that alkyl gallate with a C_7_ side chain is the best hit for the further development of a FtsZ specific antibacterial with minimal effects on overall membrane integrity.

**Table 1:** **Structures of the alkyl gallates and their activity against *B. subtilis* 168**.

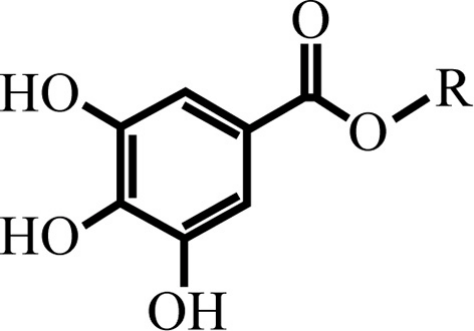					
**C_7_H_5_O_5_-R**		**R**	**MIC^a^_90_ (μg/mL)**	**MIC^a^_50_ (μg/mL)**	**MIC^b^_24H_ (μg/mL)**
Compound 8	Pentyl gallate	(CH_2_)_4_CH_3_	235	141	400
Compound 9	Hexyl gallate	(CH_2_)_5_CH_3_	90	65	100
Compound 10	Heptyl gallate	(CH_2_)_6_CH_3_	50	20	50
Compound 11	Octyl gallate	(CH_2_)_7_CH_3_	105	75	25

a *Determined by the REMA assay*.

b *Determined as minimum concentration that shows no growth after 24 h in a 2-fold dilution series*.

## Materials and methods

### General

DNA manipulations including molecular cloning in *Escherichia coli* DH5α, PCR, DNA sequencing, restriction, ligation, and transformation were performed using standard methods (Sambrook et al., 1989). Restriction enzymes, T4 DNA Ligase and *Phusion* DNA polymerase were used as specified by the supplier (Fermentas). Both *E. coli* and *B. subtilis* were grown at 37°C on solid medium (LB Lennox plus agar 1.5% w/v) (Sezonov et al., 2007), and liquid medium (LB Lennox). When appropriate, ampicillin and spectinomycin were added to final concentrations of 100 and 50 μg/mL, respectively. Starch (Sigma Aldrich) was used at 0.1%. Primers are listed in Table 2. Plasmids and strains are listed in Table 3. *B*. *subtilis* 168 genomic DNA was isolated using the Wizard genomic DNA kit (Promega) according to the suppliers' instructions.

**Table 2:** **Primers used for cloning**.

**Name**	**Sequence (5′-3′)**	**Location**
AB7	GCAGC**GCTAGC**ATTACTTGTACAGCTCGTCCATGCCGAG	Reverse primer for *eyfp* with *Nhe*I site
AB10	GCCGCA**GAATTC**ATGTTGGAGTTCGAAAC	Forward primer for *ftsZ* with *EcoR*I site
AB55	TAGCAT**GGATCC**GGCGGCGGCGGCTCCGGTGGTGGTGGTTCCGGCGGCGGCGGCATG GTGAGCAAGGGCGAGG	Forward primer for *eyfp* with flexible linker sequence and *BamH*I site
AB56	TAGCAT**GGATCC**GCCGCGTTTATTACGGTTTC	Reverse primer for *ftsZ* with *BamH*I site

*Restriction sites used to clone are in bold. Initiation or termination codons are underlined*.

**Table 3:** **Plasmids and strains**.

**Name**	**Relevant characteristics**	**Source**
***PLASMIDS***		
pMK13	*ampR, bgaA', tetR, PZn-MCS-linker-yfp,'bgaA*	Kjos and Veening, unpublished
pDOW01	pDR111 derivative with pUC18 ori, *amyE::spc P_hyperspac_*	Ruud Detert Oude Weme, unpublished
pDJ108	*amyE::spc P_hyperspac_-ftsZ-eyfp*	This work
pEK5	*lacZa, trc HisTag-enterokinase site-ezrA27-562*	(Krol et al., 2012)
***B. subtilis STRAINS***		
168	*trpC2*	Bacillus Genetic Stock Center (www.bgsc.org)
4055	*trpC2 amyE::spc P_hyperspac_-ftsZ-eyfp*	This work
***E. coli STRAINS***		
DH5α		Life Technologies

*Spc, Spectinomycin resistance*.

### Plasmid and strain construction

To generate vector pDJ108, a PCR product containing the coding sequence of *eyfp* (enhanced Yellow Fluorescent Protein) was amplified from vector pMK13, using primer AB7/AB55 (Table 2). The PCR product was sequenced and subsequently digested with *BamH*I/*Nhe*I. *ftsZ* was amplified from the genomic DNA of *B*. *subtilis* 168 using primer AB10/AB56 (Table 2). The PCR product was sequenced and subsequently digested with *EcoR*I/*BamH*I, and ligated with the digested *eyfp* PCR product and *EcoR*I/*Nhe*I digested pDOW01 resulting in plasmid pDJ108, which was verified by sequencing. Subsequently, *B. subtilis* 168 was transformed with pDJ108, and correct integration at the *amyE* locus was verified on starch plates (Harwood and Cutting, 1990). A positive colony was selected, expression and localization of the fusion protein FtsZ-eYFP was verified by microscopy (as described below for *in vivo* Z-ring analysis) resulting in strain 4055.

### Alkyl gallates (compounds 8–10)

Alkyl gallates with side chains varying from five to eight carbons [pentyl (compound 8), hexyl (compound 9), heptyl (compound 10), and octyl (compound 11) gallates] were synthetized as described (Silva et al., 2013). Compound numbers were chosen to keep in line with our previous report (Silva et al., 2013).

### MIC assays

#### REMA

The antibacterial activity of alkyl gallates was tested by the standard resazurin microtiter assay (REMA) plate method with some modifications (Palomino et al., 2002; Silva et al., 2013). Briefly, an overnight culture of *B. subtilis* was grown in 5 mL LB medium. The overnight culture was diluted with LB medium and distributed into a 96-well microtiter plate to a final volume per well of 100 μL (10^5^ CFU/well). The alkyl gallates were serially diluted with LB (1000–15 μg/mL), and 100 μL dilutions were added to the 96-well microtiter plate. Kanamycin (50 μg/mL) was used as control antibiotic. The plate was subsequently incubated at 37°C for 4 h. After 4 h, 15 μL of a 0.01% (wt/vol) freshly prepared resazurin solution in H_2_O was added to each well and the plate was incubated for 1 h. Subsequently, the fluorescence in each well was measured in a Synergy Mix Microplate Reader (BioTek), with excitation and emission wavelengths at 530 and 590 nm, respectively. All the experiments were done in triplicate and MIC_50_ and MIC_90_ values were calculated using a nonlinear regression approach (Silva et al., 2013).

#### Dilution

Cells from an exponentially growing *B. subtilis* culture were inoculated at OD_600_ of 0.2 in LB medium, at 37°C, with alkyl gallates in 2-fold dilution series. After 24 h, the lowest alkyl gallate concentration at which no visible growth occurred was scored as MIC_24H_.

### General microscopy analysis

Cells were resuspended in small volumes of CH medium (Sharpe et al., 1998) or Phosphate Buffered Saline (PBS, 58 mM Na_2_HPO_4_; 17 mM NaH_2_PO_4_; 68 mM NaCl, pH 7.3), and mounted on an agarose pad (1% w/v in PBS). Cells were imaged using a Nikon Ti-E microscope (Nikon Instruments, Tokyo, Japan) equipped with a Hamamatsu Orca Flash4.0 camera. Image analysis was performed using the software packages ImageJ (http://rsb.info.nih.gov/ij/) and Adobe Photoshop (Adobe Systems Inc., San Jose, CA, USA).

### *In vivo* Z-ring analysis

An overnight culture of *B. subtilis* 4055 grown in LB with spectinomycin at 37°C was diluted 1:100 into fresh LB with 0.02 mM IPTG to express *ftsZ-eyfp*. When the culture reached an OD_600_ of 0.4, 1 mL culture was centrifuged (1 min, 22,000 *g*), resuspended in 100 μl LB, and incubated at 37°C in the presence of either dimethyl sulfoxide (DMSO, 2% v/v), nisin (1.5 μg/mL), carbonyl cyanide m-chlorophenyl hydrazone (CCCP, 0.2 mM) or alkyl gallates at MIC_50_ concentrations (see Table 3). After either 2 min or 15 min of incubation, the cells were collected (1 min, 22,000 × *g*), resuspended in 50 μl of PBS and mounted on agarose pads for microscopy analysis.

### Brightfield microscopy

An overnight culture of *B. subtilis* 168 grown in LB at 37°C was diluted 1:100 into fresh LB until early exponential phase, OD_600_ of 0.3. 3 mL samples of the culture were taken and incubated at 37°C in the presence of 2 μg/mL 3-[(6-chlorothiazolo[5,4-b]pyridin-2-yl)methoxy]-2,6-difluorobenzamide (PC190723, Merck), and alkyl gallates at various concentrations. After 1h, 2h, or 3h of continued growth in the presence of the compounds, a sample of 1 mL was collected (1 min, 22,000 × *g*) and cells were fixed by the addition of 1 mL of 8% formaldehyde and incubation at 23°C for 60 min. Subsequent to fixation, cells were washed twice in PBS and resuspended in 10–30 μL of PBS before mounting on agarose pads for microscopy analysis.

### Membrane permeability assay

An overnight culture of *B. subtilis* 168 grown in LB was diluted 1:100 into fresh LB and grown at 37°C until exponential phase, OD_600_ of 0.5. A sample of 1 mL was collected and cells were resuspended in 50 μL of CH medium (Sharpe et al., 1998). Membrane integrity was assessed using the commercial assay Live/Dead BacLight bacterial viability kit (Invitrogen) for microscopy, according to the manufacturer's instructions. The dyes propidium iodide (20 mM) and SYTO 9 (3.34 mM) were combined in equal amounts and 0.15 μL of the dye mixture was used to stain the DNA of cells resuspended in 50 μL of CH medium. Cells were incubated for 15 min. at 23°C in the presence of DMSO (2%), Nisin (2.5 μg/mL), CCCP (0.2 mM) and alkyl gallates at MIC_50_ and MIC_90_ (see Table 3). After incubation, cells were mounted on agarose pads for microscopy analysis and the green and red fluorescence were imaged. In total, two independent experiments were performed. Per experiment, more than 250 cells for each condition were scored for green (intact membrane) or red (permeabilized membrane) fluorescence. The values were converted into percentages and the means and standard deviations were plotted on a graph using Excel.

### Protein expression and purification

*B. subtilis* FtsZ was expressed and purified using the ammonium sulfate precipitation method as described before (Mukherjee and Lutkenhaus, 1998; Krol and Scheffers, 2013). His-EzrA_cyt_ was expressed as described (Krol et al., 2012). For purification, pellet from one liter of culture was resuspended in 20 mL of Buffer A (50 mM tris(hydroxymethyl)aminomethane [Tris]/HCl pH = 7.5, 250 mM NaCl, 10 mM imidazole) supplemented with a EDTA (ethylenediaminetetraacetic acid)-free protease inhibitor tablet (Roche). The cells were disrupted at 18 kpsi (Constant systems OneShot disruptor, LA biosystems) and the insoluble fraction was removed by centrifugation at 7000 xg for 20 m at 4°C. Supernatant was applied onto Ni-NTA Agarose resin (Qiagen). The resin was washed with Buffer A containing 25 mM imidazole and His-EzrA_cyt_ protein was eluted with the same buffer containing 300 mM imidazole. Samples were dialyzed and stored at −80°C in a buffer containing 20 mM Tris/HCl, pH = 7.5, 250 mM NaCl, 10% glycerol.

### GTPase assay

The FtsZ GTP hydrolysis rate was determined using the malachite green phosphate assay described in Krol and Scheffers (2013) with the following modifications. Two fold concentrated stocks of alkyl gallates with FtsZ were prepared in polymerization buffer (50 mM 4-(2-hydroxyethyl)-1-piperazineethanesulfonic acid [Hepes]/NaOH, pH = 7.5; 300 mM KCl; 0.02% Triton X-100) and incubated for 5 min at 30°C. After that, 2 mM GTP dissolved in 50 mM Hepes/NaOH, pH = 7.5; 300 mM KCl was added. The final concentrations of GTP and Triton X-100 in the sample were 1 mM and 0.01%, respectively.

### Binding of alkyl gallates to FtsZ

Binding of alkyl gallates to FtsZ was assessed by monitoring the increase of alkyl gallate fluorescence upon binding to protein, analogous to Takai et al. (2011). Alkyl gallates at constant concentration were incubated with increasing amounts of FtsZ (0.6–14.4 μM). After addition of protein, the solution was incubated for 3 min at room temperature to allow equilibration of binding. Fluorescence was excited at 271 nm and emission spectra (320–450 nm) were acquired in a QuantaMaster™ spectrofluorometer controlled by the FelixGX program (Photon Technology International, Inc.). The fluorescence spectra of corresponding blanks resulting from titration of FtsZ into buffer alone were recorded and subtracted from the respective data sets. All measurements were done in polymerization buffer: 50 mM Hepes/NaOH pH = 7.5, 50 mM KCl in the absence of GTP. The change in fluorescence of alkyl gallates upon binding to FtsZ was used to determine the dissociation constant (K_d_) of the interaction between the compounds and FtsZ as described in Kelley et al. (2012). The dissociation constant was determined using MATLAB (The MathWorks, Inc., Massachusetts, U.S.A.).

### FtsZ sedimentation assays

FtsZ (10 μM) was mixed with the alkyl gallates (or an equal volume of DMSO as control) at 50 or 100 μg/mL in the polymerization buffer (50 mM Hepes/ NaOH, pH = 7.5, 50 mM KCl) supplemented with 10 mM MgCl_2_. After incubation for 5 min at 30°C, an equal volume of GTP, GDP or polymerization buffer was added (final concentration of nucleotides 2 mM). The samples were incubated for another 20 min and centrifuged at 186,000 x*g* or at 350,000 x*g* when indicated for 10 min at 25°C. Pellet and supernatant fractions were analyzed by sodium dodecyl sulfate–poly-acrylamide gel electrophoresis (SDS-PAGE) as described (Krol and Scheffers, 2013). FtsZ interacting protein His-EzrA_cyt_ and bovine serum albumin (BSA) (both at 10 μM) were used as controls.

### Electron microscopy

Electron microscopy samples were prepared essentially as described for the sedimentation assays. FtsZ (10 μM) was mixed with alkyl gallates at 50 μg/mL in polymerization buffer (50 mM Hepes/ NaOH, pH = 7.5, 50 mM KCl, 10 mM MgCl_2_), incubated for 5 min at 30°C with shaking (300 rpm), after which GTP was added to a final concentration of 2 mM. The samples were incubated at 30°C for another 20 min and the polymerization mixture was applied to an electron microscopy grid as described in Krol and Scheffers (2013). To test the effect of alkyl gallates on pre-polymerized FtsZ, the protein was incubated at 30°C in polymerization buffer (25 mM piperazine-N,N′-bis(2-ethanesulfonic acid) [PIPES]/NaOH pH = 6.8, 300 mM KCl, 10 mM MgCl_2_) and polymerized for 2 min with 2 mM GTP—this results in maximal polymerization as determined by light scattering (Krol and Scheffers, 2013). After 2 min, alkyl gallates (50 μg/mL) were added and the samples were incubated for another 6 minSamples were collected at two times, immediately after alkyl gallates were added and after 6 min of incubation with alkyl gallates, and grids were prepared as described in Krol and Scheffers (2013). The grids were examined in a Philips CM120 electron microscope equipped with a LaB_6_ filament operating at 120 kV. Images were recorded with a Gatan 4000 SP 4 K slow-scan CCD camera at 36,400 × magnification.

## Results

### Alkyl gallates with carbon side chain length C_5_–C_8_ inhibit *B. subtilis* growth

Recently, we showed that pentyl (compound 8), hexyl (compound 9), heptyl (compound 10), and octyl (compound 11) gallates disrupt cell division in Xac. Delocalization of GFP-ZapA from the cell division site suggested that the FtsZ-ring is a target of these compounds (Silva et al., 2013). To further study the mode of action of these compounds we tried to overexpress and purify Xac FtsZ, but failed to do so (data not shown). Therefore, we chose to use *B. subtilis* FtsZ for a more detailed characterization of the mechanism of action of alkyl gallates. First, we tested the antibacterial activity of compounds 8–11 against *B. subtilis* 168, using the REMA assay. This assay determines the concentrations at which compounds block bacterial metabolic activity over a period of 4 h. Using this assay, these compounds were found to block cell activity with MIC_90_ concentrations ranging from 50 to 235 μg/mL (Table 1). In the REMA assay, compound 10 (C_7_) was the most potent (Table 1). In an earlier report, Kubo and co-authors found similar MIC values for compounds 9 and 10, and a lower MIC for compound 11 against *B. subtilis* strain ATCC 9372 (12.5 μg/mL) using the macrodilution method (Kubo et al., 2004). Since compound 10 was the most potent against *B. subtilis* and alkyl gallates with longer and shorter side chains than compounds 8–11 did not disrupt septum formation in Xac (Silva et al., 2013), we focused on compounds 8–11 in our study.

### Alkyl gallates disrupt Z-ring formation *in vivo*

**Figure 1:**
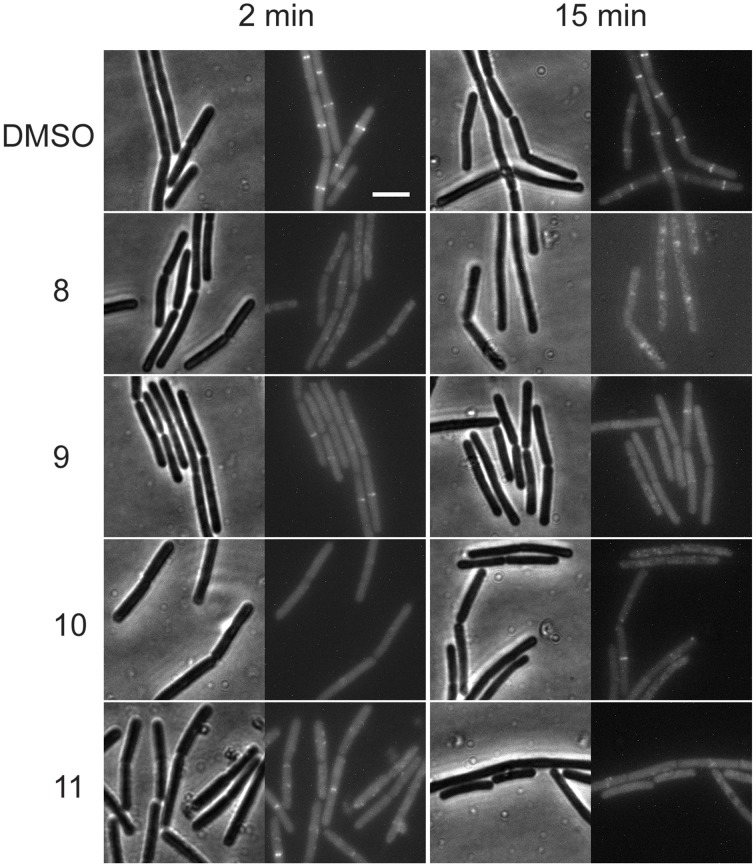
**Alkyl gallates disrupt the Z-ring**. *B. subtilis* cells expressing *ftsZ-eyfp* were incubated with DMSO (2%) or alkyl gallate compounds at MIC_50_ for 2 min (left columns) or 15 min (right columns). Brightfield and fluorescence microscopy images are shown. Incubation with alkyl gallates led to the disappearance of Z-rings and increase of fluorescence in the cytoplasm. Scale bar (same for all): 5 μm.

To study whether FtsZ ring formation is the target of the alkyl gallates, we used a *B. subtilis* strain (4055) that expresses an additional copy of *ftsZ* fused to the fluorescent protein eYFP from the ectopic *amyE* locus. In this strain, the FtsZ-eYFP fusion protein localized to the division site at mid-cell (Figure 1) and mid-cell localization was not affected by DMSO, which was used as a solvent for the compounds. Incubation with alkyl-gallates at MIC_50_ interfered with the formation or stability of the Z-ring. After 2 min of incubation, the FtsZ-eYPF fluorescence pattern was mostly found spread throughout the cell, even though some Z-rings could still be detected (most noticeably after incubation with compound 9 and compound 11). After 15 min of incubation the effect was more pronounced, with fluorescence throughout the cytosol, sometimes with occasional fluorescent spots, but hardly any Z-rings. The disruption of the Z-ring seemed to be fast, although it became more evident with an increase of incubation time. The occasional observance of rings was consistent with the fact that the cells were incubated with compounds at MIC_50_, meaning that it was expected that not every cell was affected. To confirm that the disappearance of the FtsZ rings is not caused by a generic loss of membrane integrity, the effect of membrane potential dissipation and membrane pore formation on FtsZ rings was determined using carbonyl cyanide m-chlorophenylhydrazone (CCCP) and Nisin (Strahl and Hamoen, 2010). After incubation of the strain 4055 with CCCP and nisin, various Z-rings localized at the mid-cell (Figure S1), although rings are less bright after CCCP treatment as reported before (Strahl and Hamoen, 2010). The presence of Z-rings after CCCP and nisin treatments indicate that the disappearance of the FtsZ-rings caused by the alkyl gallates is not caused by a generic loss of membrane integrity.

### Alkyl gallates inhibit GTPase activity of FtsZ

**Figure 2:**
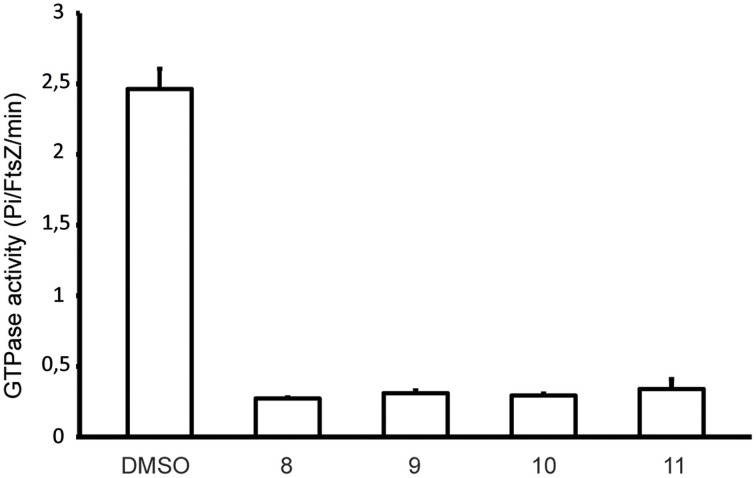
**Alkyl gallates inhibit FtsZ GTPase activity**. FtsZ GTPase activity was determined as described in Materials and Methods. Compound concentrations were 50 μg/mL, 1.5% DMSO (compound vehicle) was used as a control. Three independent experiments were performed, average and standard deviation are shown.

The disruption of the Z-ring in cells treated with alkyl gallates suggested that FtsZ is a direct target for these compounds. To test this we made use of the polymerization-associated GTP hydrolysis activity of FtsZ. GTP hydrolysis is often used to screen for FtsZ inhibitors from a compound library (Anderson et al., 2012). The GTPase activity of FtsZ in the presence of 50 μg/mL of the alkyl gallates was reduced nearly 6-fold compared to the control sample. However, residual GTPase activity was still detected in all of the samples, indicating that FtsZ was not completely inactive (Figure 2). All of the compounds showed similar levels of inhibition of the GTPase activity of FtsZ. The GTP hydrolysis assay was performed in the presence of Triton X-100 as it has been reported that some compounds identified in high-throughput screens form small aggregates that inhibit FtsZ activity non-specifically. This aspecific inhibition is abolished by the inclusion of Triton X-100 in the assay (Anderson et al., 2012). The experiment was also performed in the absence of Triton X-100 and similar results were obtained (not shown). Combined, this indicates that the effect of the alkyl gallates on FtsZ hydrolysis was specific and not caused by aggregation of the compounds.

### FtsZ is a direct target for alkyl gallates

**Figure 3:**
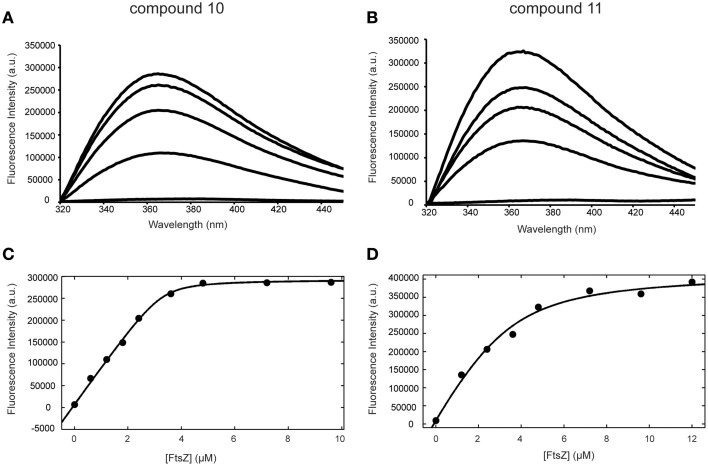
**Binding of compounds 10 and 11 to FtsZ monitored by fluorescence spectroscopy**. Fluorescence emission spectra of compound 10 **(A)** and 11 **(B)** acquired in the presence of 0, 1.2, 2.4, 3.6, 4.8 μM FtsZ (from bottom to top). **(C,D)** Binding curves of compounds 10 **(C)** and 11 **(D)** to FtsZ. The change in fluorescence intensity at 366 nm was plotted against FtsZ concentration (from 0 to 9.6 μM for compound 10 and from 0 to 12 μM for compound 11).

The binding of alkyl gallates to FtsZ was monitored using the intrinsic fluorescence of the compounds. FtsZ was titrated into a quartz cuvette containing a compound at fixed concentration (see Materials and Methods) and fluorescence emission spectra of the alkyl gallates were recorded with the excitation wavelength set at 271 nm as described in Takai et al. (2011). Compounds 10 and 11 showed strong binding to FtsZ: the fluorescence emission maximum shifted from 389 to 366 nm and the maximum signal intensity increased upon FtsZ addition (Figures 3A,B). The fluorescence of alkyl gallates was plotted as a function of FtsZ concentration and the dissociation constant was determined using 1:1 binding formalism described in Kelley et al. (2012). The best fits (*R*^2^ = 0.99) were obtained for a fixed concentration of compounds at 3.3 μM. The estimated K_d_ values obtained were 0.08 ± 0.03 μM for compound 10 and 0.84 ± 0.22 μM for compound 11 (Figures 3C,D).

Tryptophan and tyrosine fluorescence are also excited at 271 nm. However, FtsZ does not contain tryptophan and the emission maximum of tyrosine occurs at 303 nm and does not change according to solvent polarity. The emission maximum of alkyl gallates occurs at 389 nm. We observed that tyrosine emission does not change upon binding of FtsZ to compound 10 and 11. However, addition of compound 8 and 9 significantly quenched tyrosine fluorescence of FtsZ. During analysis, the fluorescence spectra obtained for the compounds in the presence of FtsZ were corrected for protein fluorescence by subtracting the spectra of samples containing only FtsZ. The change in the FtsZ emission spectra due to tyrosine quenching made proper analysis of binding of compound 8 and 9 to FtsZ very difficult (Figures S2A,B). The resulting shift in emission maximum for compounds 8 and 9 was severely reduced compared to compounds 10 and 11. For compound 8, an estimated K_d_ of 3.1 ± 2.0 μM could be calculated, with a worse fit (*R*^2^ = 0.83) than was obtained for compounds 10 and 11 (Figure S2C). For compound 9, an estimated K_d_ could not be calculated. Combined, these results show that compounds 10 and 11 specifically bind FtsZ, with compound 10 having the highest affinity. The quenching observed with compounds 8 and 9 indicates interaction of the compounds with FtsZ but the low shifts in emission maximum and the difficulties in K_d_ estimation indicate that the binding of compounds 8 and 9 to FtsZ may be aspecific.

### Sedimentation of FtsZ is enhanced in the presence of alkyl gallates

**Figure 4:**
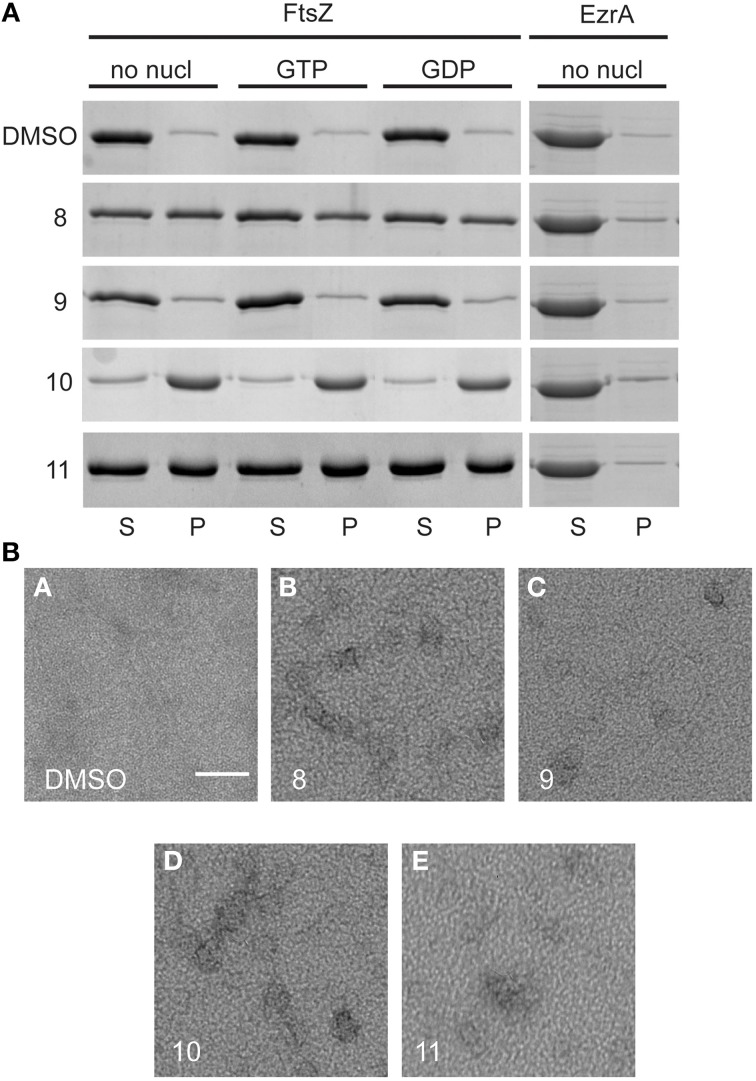
**Sedimentation of FtsZ in the presence and absence of alkyl gallates and structures formed after treatment with alkyl gallates. (A)** 10 μM FtsZ was incubated with alkyl gallates (50 μg/mL) before GTP/GDP was added. In the sample without nucleotide (no nucl), the same volume of polymerization buffer was added to the sample as for GTP/GDP. His-EzrA_cyt_ was sedimented without nucleotide. (S) indicates supernatant and (P) pellet fractions from the experiment. As a control, 1.5% DMSO was used. All experiments were performed in triplicate. **(B)** The same experiment was performed, with GTP, and structures of FtsZ were visualized by EM. Scale bar: 100 nm.

Next, we used a sedimentation assay to study the effect of alkyl gallates on the assembly of FtsZ *in vitro*. FtsZ was mixed with the alkyl gallates (at 50 μg/mL) or 1.5% DMSO and polymerization was started by addition of GTP or GDP (control) to the sample. We noticed that in the presence of compounds 8, 10, and 11, FtsZ was recovered in the pellet fraction above background levels independent on the presence and type of nucleotide used (Figure 4A). This result suggested that the compounds induce protein clustering or aggregation. As both the FtsZ interacting protein His-EzrA_cyt_ (Figure 4A) and BSA (not shown) did not sediment in the presence of the compounds, the observed FtsZ sedimentation is not the result of aspecific protein aggregation. Compound 10 has the strongest effect on FtsZ sedimentation (all FtsZ protein was present in the pellet fraction), whereas approximately 50% of FtsZ protein was recovered in the pellet fraction after treatment with compounds 8 or 11. No sedimentation was detected when FtsZ was incubated with compound 9 (Figure 4A). However, upon higher sedimentation speed (350 000 xg) or at higher compound concentration (100 μg/mL), FtsZ was recovered above background levels also with compound 9 and 100% of FtsZ was present in the pellet fraction after treatment with compounds 8, 10, and 11 (Figure S3). The strength of the effects of the various alkyl gallates on FtsZ sedimentation are in line with the estimated K_d_s for the different compounds.

The structures formed by FtsZ treated with alkyl gallates were visualized by electron microscopy (EM). As expected, FtsZ formed clusters after incubation with all the alkyl gallates (Figure 4B).

### Alkyl gallates promote clustering of FtsZ and bundling of FtsZ polymers

**Figure 5:**
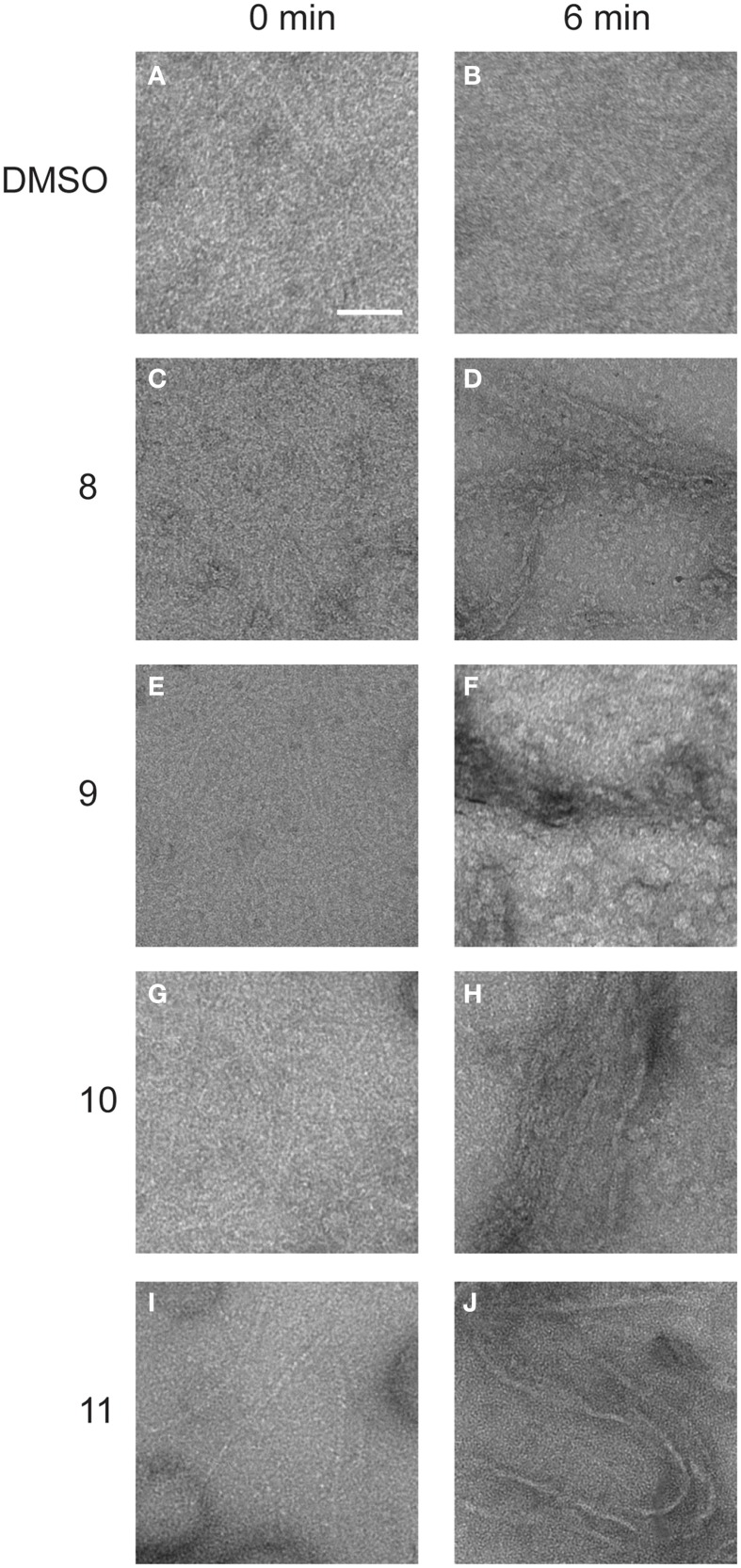
**Structures of FtsZ formed in the presence and absence of alkyl gallates**. 10 μM FtsZ was polymerized with 2 mM GTP for 2 min. After polymers were formed, alkyl gallates or DMSO (1.5% w/v) were added and immediately sample 0 was collected (0 min, **A,C,E,G,I**). After 6 min of incubation, another sample was collected (6 min, **B**: DMSO, **D**: compound 8, **F**: compound 9, **H**: compound 10, **J**: compound 11). Scale bar: 100 nm.

Alkyl gallates caused the clustering of FtsZ and only a small amount of short polymers was detectable in the sample when GTP was added. When FtsZ was incubated with the respective compounds at MIC_90_ values, only big protein clusters and aggregates were observed (Figure S4). We assume that FtsZ was not able to polymerize because the clustering of FtsZ prevents the correct association of FtsZ molecules required for polymerization. To establish whether the alkyl gallates disrupt existing polymers, we performed an experiment in which polymerization of FtsZ was initiated before the addition of compounds to the sample. FtsZ was polymerized in a PIPES/KCl buffer that ensures optimal polymerization as determined by light scattering (Krol and Scheffers, 2013), and the compounds or DMSO were added to polymerized FtsZ. Samples were collected immediately after the addition of compounds and after an additional 6 min of incubation. Compounds 8, 10, and 11 bound to the polymers of FtsZ and induced the formation of irregular bundles (Figure 5). The amount of bundles that were observed in samples treated with compounds 10 and 11 was much higher than for compound 8—although it has to be noted that this method is not quantitative. In the samples with compound 9, bundles were almost not visible (Figure 5). We could only detect a few small bundle-like structures under the conditions used. The presence of a high number of tubules in the samples with compounds 10 and 11 suggests that the compounds can easily bind to the polymeric form of FtsZ, whereas binding of compound 8 and 9 to polymers of FtsZ is less strong—again, this is in line with the overall stronger FtsZ-binding of compounds 10 and 11. Although the alkyl gallates induce the formation of clusters of FtsZ monomers that as a result no longer form polymers, the compounds do not disrupt existing FtsZ polymers.

### Membrane integrity and cell viability are affected by alkyl gallates

**Figure 6:**
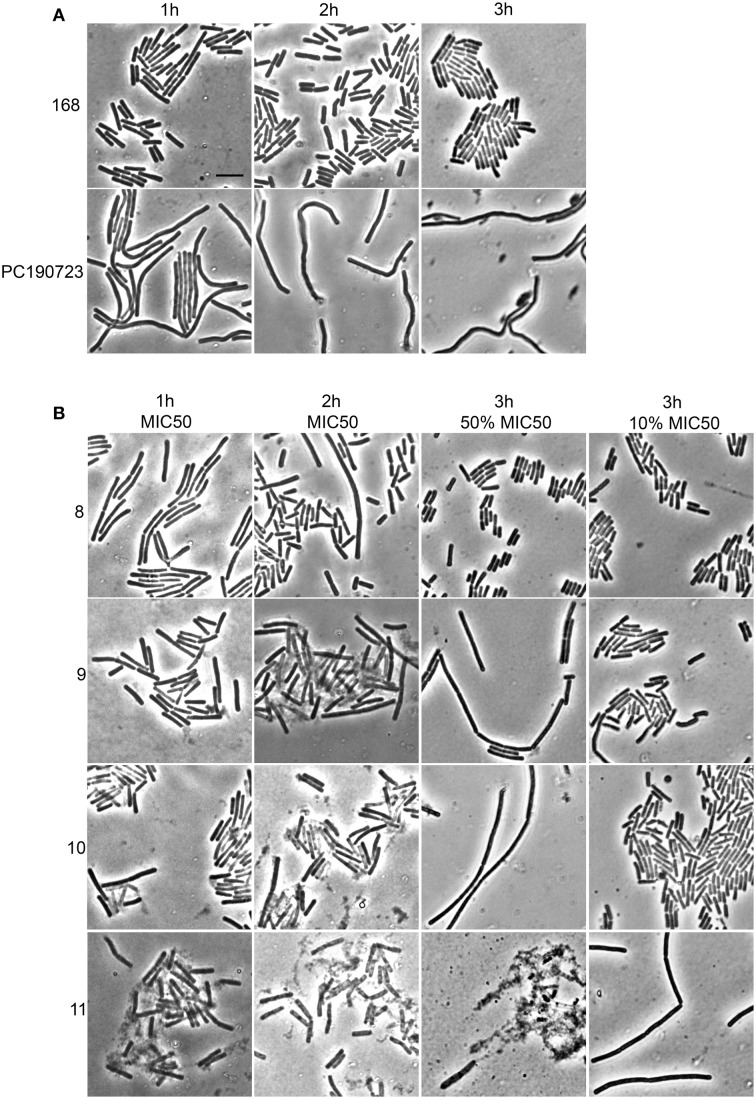
**Alkyl gallates cause cell elongation and lysis. (A)** controls: *B. subtilis* cells were incubated with nothing (168) or with PC190723 at 2 μg/mL for 1 h, 2 h, or 3 h. **(B)** *B. subtilis* cells were incubated with alkyl gallates at MIC_50_ for 1 h (1st column) or 2 h (2nd column), or for 3 h at 50 and 10% of MIC_50_ (3rd and 4th column, respectively). Brightfield microscopy images are shown. There is evident cell lysis after incubation at MIC_50_ with compounds 9, 10, and 11, whereas cell elongation is seen at concentrations below MIC_50_. Scale bar (top left, same for all): 5 μm.

Cells in which the FtsZ activity is compromised, either through mutation or by the addition of FtsZ targeting compounds, display cell elongation and filamentation caused by delayed or defective division (Addinall et al., 1996). Even though the alkyl gallates had clear and immediate effects on FtsZ rings (Figure 1), elongated cells could only occasionally be observed upon longer incubation at MIC_50_, whereas cells grown in the presence of the known FtsZ-targeting compound PC190723 (Haydon et al., 2008) were clearly filamentous (Figure 6A). After 1 h of incubation with the compounds 9, 10, and 11 at MIC_50_, we noticed that some cells had already lysed, and lysis was more noticeable when the incubation time was extended to 2 h. At MIC_90_, compounds 9, 10, and 11 caused cell death at an equivalent or higher proportion (data not shown). Incubation with compound 8 did not cause noticeable lysis—at least not to the extent as the other compounds–however, the large majority of the non-lysed cells did not appear elongated. These results indicate that the alkyl gallates can cause cell death via another mechanism than FtsZ inhibition. These alternative mechanisms may correspond to the ones reported by the REMA assay for antibacterial activity, as elongation still requires metabolic activity. Therefore, we investigated the effects of the alkyl gallates at lower concentrations than the MIC_50_, determined in the REMA assay. Lowering the concentrations of the compounds revealed that compounds 9, 10, and 11 are capable of causing cell elongation, as expected for FtsZ inhibitors, whereas compound 8 had no effect on cell length (Figure 6B). The minimal inhibitory activity (MIC_24H_) of the compounds was established by a 2-fold dilution series in which prolonged incubation revealed that compound 11 can prevent cell growth at a lower concentration than the one determined by the REMA assay (25 μg/mL Table 1). This is closer to the value reported in the literature for this compound (Kubo et al., 2004). Compounds 8, 9, and 10 show similar inhibition of growth after 24 h as previously determined by the REMA assay. Taken together these results indicate that alkyl gallates with a high affinity for FtsZ *in vitro* can induce cell death by directly targeting FtsZ. Additionaly it is observed that, at higher concentrations of the alkyl gallates an alternative mechanism is responsible for the quick cell death that takes place without cell elongation (Figure 6B).

**Figure 7:**
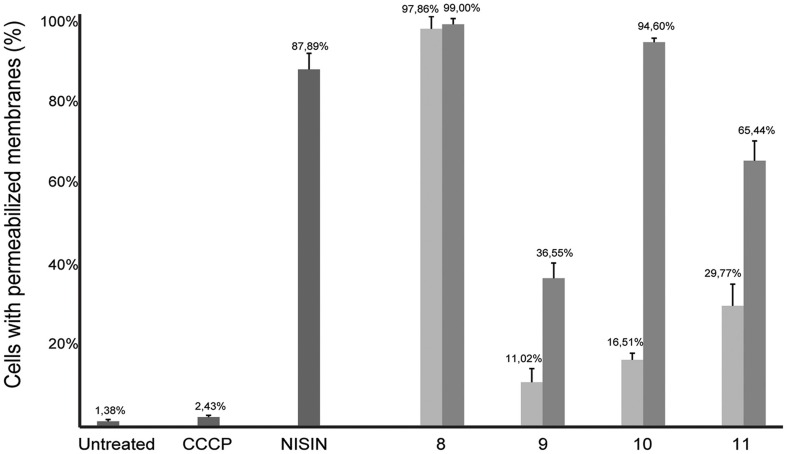
**Membrane integrity is affected in the presence of alkyl gallates**. *B. subtilis* cells were incubated with alkyl gallates, at MIC_50_ (light gray bars) and MIC_90_ (dark gray bars), and with CCCP (2 mM) or nisin (2.5 μg/mL) for 15 min, and membrane integrity was monitored using the Live/Dead assay (see text). The experiment was performed twice and more than 250 cells per incubation were counted per experiment. The values were converted to percentages, average percentage and standard deviation are shown.

As several described FtsZ inhibitors also affect cell membranes (Foss et al., 2013), we decided to study the effect of the alkyl gallates on cell membrane integrity *in vivo*. The Live/Dead BacLight kit, which combines the green membrane permeable fluorescent DNA dye, SYTO 9, and the red membrane impermeable fluorescent DNA dye, propidium iodide, was used to assess membrane integrity. Cells were incubated with both dyes and alkyl gallates. Subsequently, cells were imaged and red or orange cells were classified as cells with affected membrane integrity, whereas green cells were classified as cells with intact membranes. Nisin, which is known to make pores in the membrane, and CCCP, which disrupts the membrane potential but does not make pores (Strahl and Hamoen, 2010), were used as controls. All alkyl gallates were found to be able to create membrane pores *in vivo*, to different extents (Figure 7). Compound 8, at both MIC_50_ and MIC_90_ concentrations, permeabilized all cells, which is in line with the observed cell death without elongation—although it has to be noted that cells incubated with compound 8 at MIC_50_ did not noticeably lyse (Figure 6). Compounds 9, 10, and 11 showed a concentration-dependent membrane permeabilization—but importantly, incubation at MIC_50_ concentrations never resulted in more than 50% permeabilized cells, indicating that the alkyl gallates function by targeting membrane integrity, FtsZ function, and possibly other mechanisms.

## Discussion

The use of alkyl gallates as anti-bacterial agents has been proposed in various studies in which the pharmacological activity of these compounds was described (Kubo et al., 2002a,b, 2003, 2004; Shibata et al., 2005). As semi-synthetic compounds that are derived from gallic acid, a plant metabolite, these compounds could be interesting and environmental-friendly alternatives for the control of bacterial infections of agricultural crops. To this end, we previously characterized the activity of alkyl gallates against the important citrus pathogen *X. citri* subsp *citri* (Xac) (Silva et al., 2013). Our initial studies indicated that the alkyl gallates disrupt cell division in Xac, possibly by targeting the FtsZ-ring, which is different from the observed mechanism of membrane binding that is also described for these compounds (Takai et al., 2011). Here, we investigated the mode of action of four alkyl gallates in more detail.

As we neither had access to a Xac strain expressing fluorescent FtsZ, nor were able to purify Xac FtsZ, we turned to *B. subtilis*, which is also killed by alkyl gallates (Kubo et al., 2004). Here, we provide both *in vivo* and *in vitro* evidence that FtsZ is a target for the alkyl gallates. The addition of alkyl gallates to cells expressing a fluorescent variant of FtsZ causes immediate disruption of Z-rings (Figure 1), and cultures exposed to compounds 9, 10, and 11 display the classical elongation phenotype of cells affected in cell division (Figure 6). Purified FtsZ is blocked from polymerizing in the presence of alkyl gallates (Figure 4 and Figure S4), and some of the alkyl gallates bind FtsZ with high affinity, most notable heptyl gallate with an estimated K_d_ of 80 nM (Figure 3). It should be noted that the effect of the alkyl gallates on *B. subtilis* indicated that FtsZ is not the sole target–the classical phenotype of cell elongation by division inhibition was observed for compounds 9, 10, and 11 (Figure 6), but all compounds also affected membrane integrity as observed with the permeability assay (Figure 7). Therefore, alkyl gallates probably promote cell death by a combination of mechanisms: FtsZ inhibition, membrane permeabilization and, possibly, another activity.

Two recent studies pointed out some issues with antibacterials that have been identified as “FtsZ-inhibitors.” The first study, by Anderson et al. (2012), showed that many compounds identified as FtsZ inhibitors in high-throughput screening assays based on FtsZ-mediated GTP hydrolysis, in fact form small aggregates that block GTP hydrolysis. The addition of Triton X-100 to these assays prevents aggregate formation and reveals normal GTP hydrolysis levels in the presence of “false-positive” compounds. The effect of alkyl gallates on GTP hydrolysis is the same, irrespective of whether Triton X-100 is present or not, indicating that alkyl-gallates are not false-positive GTP hydrolysis inhibitors (Figure 2). Also, our other *in vitro* assays show that—at least some—of the alkyl gallates bind FtsZ with high affinity and that these compounds cluster FtsZ and prevent polymerization (Figures 3, 4). Cluster formation is not caused by aspecific protein aggregation as shown by control experiments with His-EzrA_cyt_ and BSA. Combined, the *in vitro* work clearly shows that FtsZ is inhibited by the alkyl gallates. The second study, from Foss et al. (2013) indicated that many compounds, identified as FtsZ inhibitors, target the membrane. Alkyl gallates have already been identified as membrane binding agents (Takai et al., 2011)—therefore we examined the effect of the alkyl gallates on membrane integrity. Intriguingly, compound 8, with the shortest alkyl chain length, had the most disruptive effect on membranes as monitored by the influx of propidium iodide into cells (Figure 7). The other compounds also affected membrane integrity albeit to different extents. We observed that compounds 9–11 permeabilize less than 50% of the cells at concentrations where 50% of the cells are metabolically inactive (MIC_50_) as determined by the REMA assay. At MIC_50_, cell elongation cannot be observed and many cells in the culture lyse. At lower concentrations of the compounds, elongation can clearly be observed, and it is evident, from the dilution series (Table 1), that concentrations that cause elongation are sufficient to inhibit cell growth. We conclude that, especially for compounds 10 and 11, FtsZ inhibition occurs at lower concentrations than MICs determined with the REMA assay. Increasing the concentration to MIC values leads to disruption of membrane integrity—this is particularly obvious for compound 8. The effect of alkyl gallates on membrane integrity is not the cause for FtsZ ring disruption as compounds that disrupt membrane integrity or that dissipate the membrane potential do not affect the Z-ring *in vivo*, whereas all alkyl gallates, at MIC_50_ and MIC_90_, quickly disrupt most of the Z-rings in cells. Combined, our experiments show that alkyl gallates, in addition to targeting membrane integrity (Haydon et al., 2008; Takai et al., 2011), can also directly target FtsZ. We consider it very likely that both membrane disruption and FtsZ inhibition by alkyl gallates apply to many bacteria because: (1) alkyl gallates have a wide spectrum of antibacterial activity against both Gram-positive and Gram-negative bacteria (Kubo et al., 2002a,b, 2004; Shibata et al., 2005; Silva et al., 2013); (2) alkyl gallates disrupt bacterial model membranes (Takai et al., 2011) as well as *B. subtilis* membranes (this work); (3) a cell division phenotype was described both for the Gram-negative *X. citri* subsp. *citri* (Silva et al., 2013) as well as for Gram-positive *B. subtilis* (this work); (4) FtsZ is directly inhibited by alkyl gallates (this work)—FtsZ is highly conserved and the absence of ZapA rings described for *X. citri* subsp. *citri* (Silva et al., 2013) strongly suggests that Z-ring formation is blocked in this organism as well. Heptyl-gallate (compound 10) was found to bind to FtsZ with very high affinity, resulting in blocked cell division at low concentrations and FtsZ cluster formation *in vitro*. The length of the alkyl chains affects both the interaction with the membrane and with FtsZ. As the most promising anti-FtsZ agent, heptyl gallate can be used as a hit for the design of innovative compounds that have enhanced specificity toward FtsZ and less activity on the membrane. Several modifications on the structure of heptyl gallate are currently being made in our laboratories.

### Conflict of interest statement

The authors declare that the research was conducted in the absence of any commercial or financial relationships that could be construed as a potential conflict of interest.
