# Supplementary figures and images for: Antibacterial activity of alkyl gallates is a combination of direct targeting of FtsZ and permeabilization of bacterial membranes
Source: Front Microbiol. 2015 Apr 29;6:390. doi: 10.3389/fmicb.2015.00390 (PMC4413848; doi:10.3389/fmicb.2015.00390)

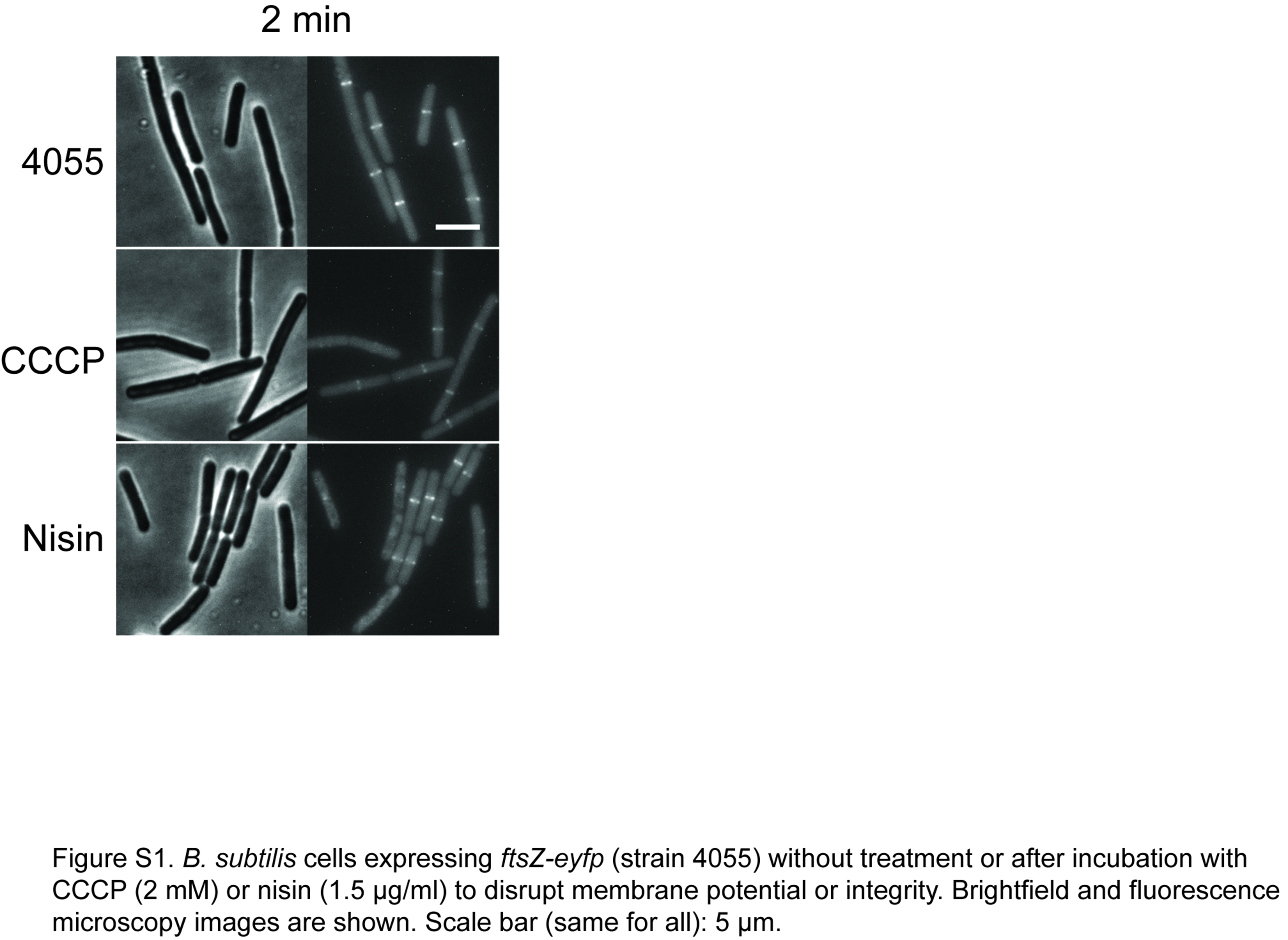

Supplement: Supplementary file 1 [file Image1.TIF]

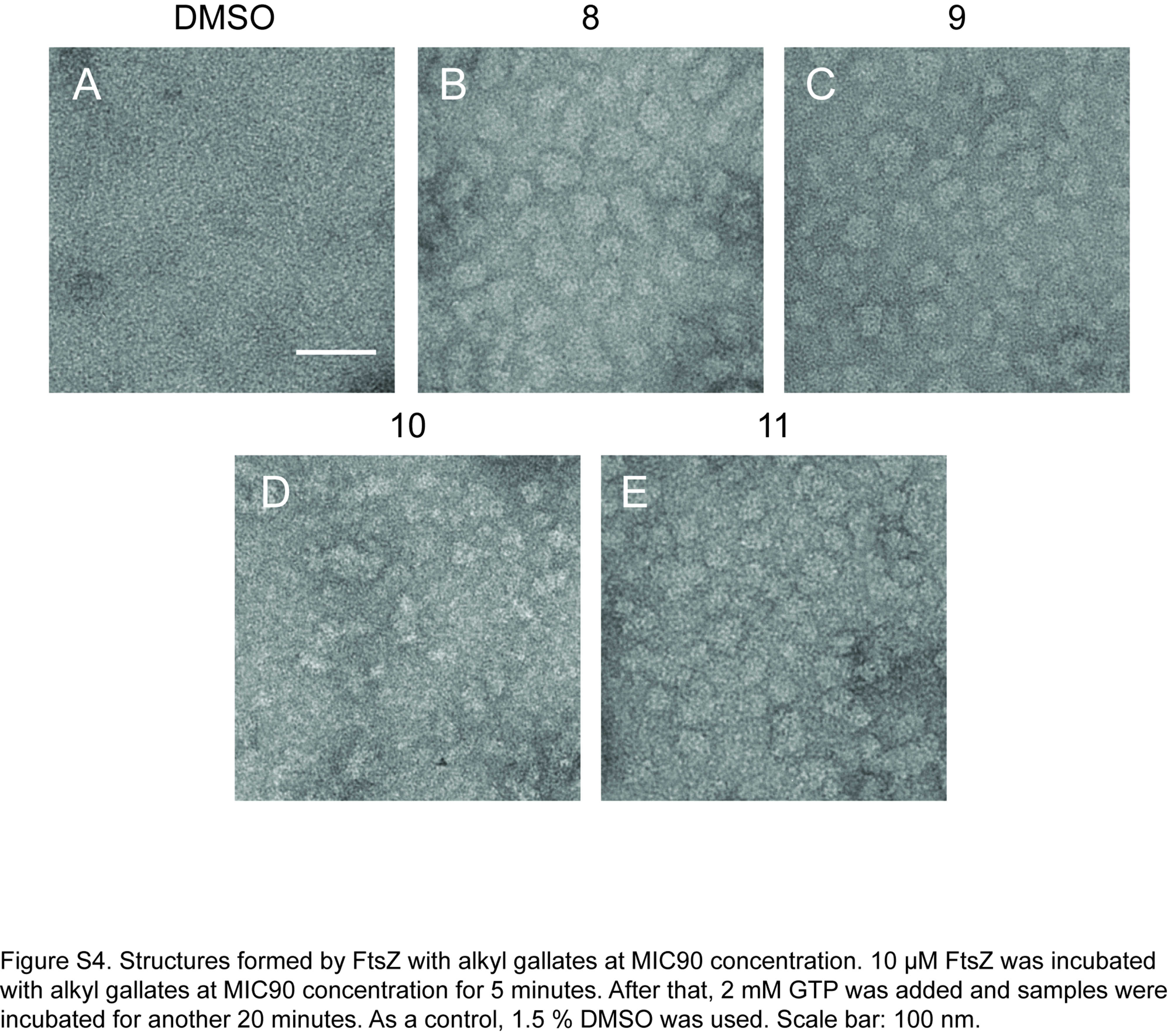

Supplement: Supplementary file 4 [file Image4.TIF]
